# Rethinking Mechanical Ventilation: Can Ventilation Mode Influence Long-Term Cognitive Outcomes in ICU Patients with COVID-19?

**DOI:** 10.3390/jcm15020898

**Published:** 2026-01-22

**Authors:** Clementina M. van Rijn, Marta Godoy-González, Sol Fernández-Gonzalo, Pierre Souren, Malcolm G. Coulthard, David J. Howard, Marijtje L. A. Jongsma

**Affiliations:** 1 Donders Institute for Brain, Cognition and Behaviour, Radboud University, 6525 GD Nijmegen, The Netherlands; 2 The Exovent Development Group, Wraysbury TW19 5JF, UK; malcolm.coulthard@nhs.net (M.G.C.); davidjhoward10@gmail.com (D.J.H.); 3 Critical Care Department, Hospital Universitari Parc Taulí, Universitat Autònoma de Barcelona, Institut d’Investigació i Innovació Parc Taulí (I3PT-CERCA), 08208 Sabadell, Spain; mrgodoy@tauli.cat (M.G.-G.); msfernandez@tauli.cat (S.F.-G.); 4 Centro de Investigación Biomédica en Red de Enfermedades Respiratorias (CIBERES), Instituto de Salud Carlos III, 28029 Madrid, Spain; 5 Centro de Investigación Biomédica en Red de Salud Mental (CIBERSAM), Instituto de Salud Carlos III, 28029 Madrid, Spain; 6 Research Technical Support Group, Department of Psychology, Radboud University, 6525 GD Nijmegen, The Netherlands; steen.souren@ru.nl; 7 Translational and Clinical Research Institute, Newcastle University, Newcastle upon Tyne NE1 7RU, UK; 8 Imperial and UCLH Trust Hospitals, London NW1 2BU, UK; 9 Behavioural Science Institute, Radboud University, 6500 HE Nijmegen, The Netherlands; marijtje.jongsma@ru.nl; 10 Faculty of Psychology, Universitas Gadjah Mada, Yogyakarta 55281, Indonesia

**Keywords:** intensive care unit (ICU), cognitive functioning, ICU-associated cognitive impairment, COVID-19, invasive mechanical ventilation (IMV), tracheostomy, positive pressure ventilation (PPV), negative pressure ventilation (NPV)

## Abstract

**Background:** Long-term cognitive impairment is common among ICU patients who required invasive mechanical ventilation (IMV). Its etiology is likely multifactorial. This preregistered study examined the association between the duration of IMV and cognitive function post-ICU, as well as the moderating effects of age and cognitive reserve. **Methods:** A secondary analysis was conducted using data from a published study of COVID-19 ICU survivors. One year after discharge, participants underwent a neuropsychological assessment. Linear regression models were used to evaluate associations between the variables. **Results:** Among patients who received IMV via endotracheal intubation, ventilation duration was not significantly associated with cognitive performance. In contrast, among tracheostomized patients, longer IMV duration was associated with better cognitive outcomes (Cohen’s *f*^2^ = 0.21). Age had a small negative main effect; in combination with IMV duration, *f*^2^ increased to 0.31. Cognitive reserve showed a strong positive association with cognitive outcome; in combination with IMV duration, *f*^2^ increased to 0.67. The interaction terms were negligible in both cases. **Conclusions:** We hypothesize that, compared to endotracheal intubation, IMV via tracheostoma may not only reduce the need for sedation, but also provide a more efficient respiratory support, therefore contributing to positive cognitive outcomes. However, IMV via tracheostomy still represents a form of positive pressure ventilation (PPV), which carries risks, such as ventilator-induced lung injury and reduced cardiac output and brain perfusion. These concerns about PPV, combined with our findings, indicate that alternative, non-invasive modes, such as negative pressure ventilation (NPV), warrant evaluation in future trials.

## 1. Introduction

We hereby present the results of a secondary, preregistered analysis of data on cognition in survivors of COVID-19, assessed one year after discharge from the intensive care unit (ICU) [1,2]. The primary objective of the present study was to identify conditions related to invasive mechanical ventilation (IMV) that may contribute to cognitive impairment.

Even long after discharge from the hospital, many former ICU patients continue to experience central nervous system symptoms, especially cognitive impairment [3,4,5]. The pathophysiology underlying post-ICU cognitive impairment is highly complex and not fully understood [5]. Multiple interacting factors may contribute to these long-term cognitive outcomes [6].

First, the underlying disease itself can have a significant impact on cognitive function. In this context, Araújo et al. reported that cognitive impairment associated with COVID-19, a virus disease known to affect multiple organ systems, including the brain [7], is not limited to hospitalized patients [8]. This finding is particularly relevant to the present study, as all participants were individuals who had recovered from COVID-19.

In addition, sedation, often needed to allow intubation in patients requiring invasive mechanical ventilation (IMV), as in our patients group, may be a major risk factor for cognitive impairment [9]. In this regard, the co-administration of multiple drugs, such as anesthetics, sedatives, and muscle relaxants [10], is a risk factor for evoking delirium, which is also thought to be a risk factor for long-term cognitive deficits [11,12,13,14].

In the present paper we focused on the *duration* of IMV. During the COVID-19 pandemic, many patients received prolonged IMV. Recent papers emphasize that ventilator-associated brain injury (VABI), resulting from various harmful factors associated with IMV, needs urgent attention [15,16]. Several factors are mentioned, among which are injurious neurosignalling caused by mechanical lung stress; inflammatory markers, probably resulting from the systemic inflammatory processes entering the brain via the circulation [15,17]; and reduced cerebral perfusion resulting from impaired cardiac output [15,17,18].

In this study, we aimed to investigate the extent to which the duration of IMV is associated with cognitive outcomes. The data used were previously collected and published in [2], where a different research question was addressed: the relationship between objective and subjective cognitive measures in COVID-19 survivors.

Beyond examining the effects of the duration of IMV on long-term cognition, we also aimed to explore the moderating role of age. Although age-corrected norm scores were used in the analyses [2], an additional moderating effect of age on the relation between ventilation duration and cognitive outcome may be present. For example, a European study from 1998 showed that, apart from duration of anesthesia, older age was a risk factor for postoperative cognitive dysfunction [19]. In line with that study, a recent prospective study by Pennings et al. [20] showed that higher total exposure to surgery requiring general anesthesia negatively impacted cognitive functioning. In their study, age was also a primary factor impacting cognitive decline. However, as anesthesia is needed for surgery, the role of anesthesia alone in post-surgical cognitive decline remains a subject of ongoing research [21,22,23]. To better understand the role of age in our patient group, we included age as a moderating factor in our analyses.

The above-cited studies [19,20] reported that education was also a factor of importance. Cognitive reserve (CogRes), a measure associated with the level of education [24], has been shown to moderate the impact of COVID-19 on cognitive functioning [25,26]. Therefore, we explored its moderating effect on the relationship between IMV duration and long-term cognitive outcomes as well.

## 2. Materials and Methods

### 2.1. Data Collection

The raw data used in the present study were provided by Marta Godoy-González, the first author of the original paper [2], and by Sol Fernández-Gonzalo, the last author. Details on recruitment of patients, the corresponding flow chart, and collection of these raw data are reported in ref. [2]. The authors of this original paper were not involved in defining the aim, research question, or analysis model for the preregistration of the present new research question. These analyses were designed by Pierre Souren, Marijtje L.A. Jongsma, Malcolm G. Coulthard, and Clementina M. van Rijn, prior to receiving any raw data [1]. The original authors became involved in the present paper only after the analyses were completed. The preregistration of the present study was published on 11 September 2024 [1]. The raw data were obtained on 23 September 2024. The data are available in Supplement S1.

### 2.2. Inspection of the Raw Data

#### 2.2.1. Variables Used

The variables used in the present analyses are listed in Table 1. Demographic and clinical data were collected retrospectively from medical records. Illness severity was assessed using the Acute Physiology and Chronic Health Evaluation II (APACHE II), and comorbidities were evaluated with the Charlson Comorbidity Index (CCI). The majority of the patients were male: 67% (55/81). Since the proportions male/female did not differ between ventilation groups (group allocation see below) (Kruskal–Wallis = 0.64, *p* = 0.70), this variable was not further considered.

At the one-year follow-up (as reported in the original study), cognitive reserve was assessed using the Cognitive Reserve Questionnaire (CRQ), a validated Spanish-language tool [2]. Total scores range from 0 to 25, with higher scores indicating greater cognitive reserve.

Cognitive performance was determined using a comprehensive neuropsychological battery. Raw scores were converted to z-scores using normative data adjusted for age and education. From these, seven cognitive domain indexes were derived [2]. These indexes were used in the present analyses. Since the z-scores of the seven cognitive domains were highly correlated (see Supplement S2.3), a composite score was calculated as follows: domain scores were first mean-centered, after which, per patient, the centered values were averaged. This individual, centered Global Cognitive Performance (GlobCogPerf) score was used as the dependent variable in the linear regression model. For the domain Processing Speed, scores were missing for three patients; their composite scores were based on six domains instead of seven (see Supplement S1).

#### 2.2.2. Group Allocation

Of the 80 patients, 29 did not receive IMV, while 51 did. Two procedures of IMV were used: with and without a tracheostoma. The duration of ventilation was considerably longer in patients who received a tracheostomy compared with those who did not. Consequently, the IMV group was divided into two ventilation groups. This subdivision of the IMV groups was not specified in the preregistration, as the submitters were not aware of these two ventilation methods at that time. Among the 51 patients who received IMV, 24 had a tracheostoma, while 26 did not. For one patient (ID 56), this information was unavailable. However, since this patient received IMV for only 2 days, it is unlikely that a tracheostoma was placed. Therefore, this patient was included in the “without tracheostoma” group, resulting in a total of *n* = 27.

### 2.3. Preregistered Analyses

**Figure 1:**
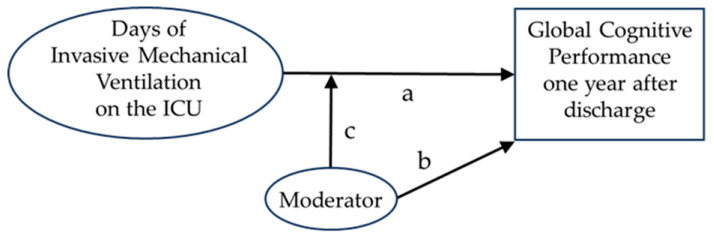
Graphical representation of the preregistered analysis.

The main research question, formulated in the preregistration, is whether the duration of IMV during ICU stay is associated with long-term cognitive functioning, operationalized as Global Cognitive Performance (GlobCogPerf), and whether such an association is moderated by the age of the patient. Additionally we aimed to explore whether the association between DoV and GlobCogPerf is moderated by the cognitive reserve (CogRes) of the patient. To address these questions, the following multiple linear regression model was specified (see also Figure 1):

GlobCogPerf = Intercept + a × DoV + b × Moderator + c × DoV × Moderator

where the variables are as follows:

- GlobCogPerf is the global cognition performance score.
- DoV is the number of days of ventilation.
- The moderator is either the patient’s age (years) or the patient’s CogRes (score)

The coefficients (slopes) are as follows:

- a: represents the relationship between DoV and GlobCogPerf (the main effect of the DoV).
- b: represents the relationship between the moderator and GlobCogPerf (the main effect of the moderator).
- c: represents the moderating effect of the moderator on the relationship between DoV and GlobCogPerf (the interaction effect between DoV and moderator).

Additionally, in accordance with the preregistration, we explored whether delirium influenced the association between DoV and GlobCogPerf. Finally, we explored separately the associations between DoV and each of the seven cognitive domains, as well as their moderation by age and by CogRes.

### 2.4. Statistics

To assess the association between DoV and GlobCogPerf, and its moderation by either age or CogRes, separate analyses were conducted for each ventilation group.

In the main analysis, age was tested as a moderator, which was our main research question. For each of the two ventilation groups, a two-step linear regression was performed: Model 1 included DoV as the sole predictor of GlobCogPerf, and Model 2 added age and the interaction term (DoV × Age) to assess moderation.

In the exploratory analysis, the same procedure was followed with CogRes as the moderator. Model 1 again included DoV only, and Model 3 added CogRes and the interaction term (DoV × CogRes). The variables were mean-centered prior to creating interaction terms.

Model fits were evaluated using the adjusted R^2^, and the improvement in explained variance between the two models was used to assess whether the addition of the moderator and the interaction term provided a better fit. Statistical significance *p* values are given in the results Section 3. Cohen’s *f*^2^ was calculated as an effect size to quantify the contribution of the additional predictors in Model 2 or Model 3 to Model 1, with values of 0.02, 0.15, and 0.35 interpreted as small, medium, and large effects, respectively. All regression analyses were performed using SPSS (version 25).

GraphPad Prism (version 7.04) was used for conducting *t*-tests, nonparametric statistical analyses, ANOVAs, and for generating the figures.

## 3. Results

The first two sections of these results describe the three groups: Section 3.1 addresses demographic and clinical characteristics, and Section 3.2 presents domain-specific cognitive performance. The subsequent sections report the analyses examining the association between GlobCogPerf and IMV duration: Section 3.3 presents the main preregistered analysis with age as a moderator, and Section 3.4 reports the preregistered exploratory analyses addressing cognitive reserve, domain-specific effects, and delirium (Section 3.4.1, Section 3.4.2 and Section 3.4.3).

### 3.1. Demographic and Clinical Variables per Group

**Figure 2:**
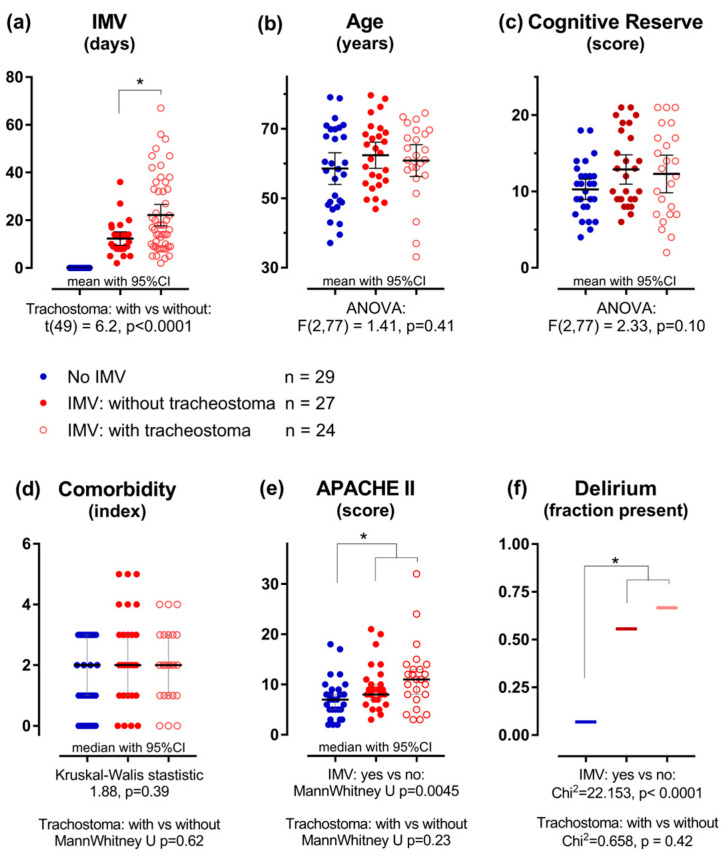
Scatter dot plots of the variables used, shown per ventilation group. The 3 groups are shown separately in the figures: Blue filled symbols: no IMV, *n* = 29; red filled symbols: IMV without tracheostoma *n* = 27; red open symbols: IMV with tracheostoma, *n* = 24. Stars (*) indicate the significant differences mentioned below. (**a**) The duration of IMV: on average, patients with a tracheostoma received IMV for a longer duration than those without: 33 days (range: 4–67) versus 12 days (range: 2–36); t(49) = 6.2, *p* < 0.0001. Age (**b**) and cognitive reserve (**c**) did not differ among the three groups. CCI (**d**) scores did not differ between the groups. The APACHE II scores (**e**) of the patients who received IMV were on average higher compared to the patients who did not: median of 9 (range: 3–32) versus median of 7 (range: 2–18); U = 460, *p* = 0.0045. (**f**) Patients that were ventilated experienced delirium more often than patients who were not ventilated: 31 of the 51 ventilated patients versus 2 of the 29 patients not ventilated (Chi^2^ = 22.2, *p* < 0.0001).

The individual data points for the demographic and clinical variables are depicted in Figure 2. Where appropriate, means, medians, or fractions are shown along with their 95% confidence intervals.

The upper three panels in Figure 2 show the independent variables, preregistered as variables of interest for the linear regression models. These variables were used in the multiple linear regression analyses, the results of which are given in the analysis section below.

The lower panels display the clinical scores reflecting the health condition of the patients during their hospital stay. These variables are of importance in the discussion. The correlation between duration of ventilation and CCI was not significant (Spearman’s ρ = 0.10, *p* = 0.50), whereas a positive correlation was found between the APACHE II score and the duration of ventilation (Spearman’s ρ = 0.41, *p* = 0.003). Figures showing correlations between the clinical scores are provided in Supplement S2.1 and S2.2.

### 3.2. Cognitive Performance (CogPerf) per Group

**Figure 3:**
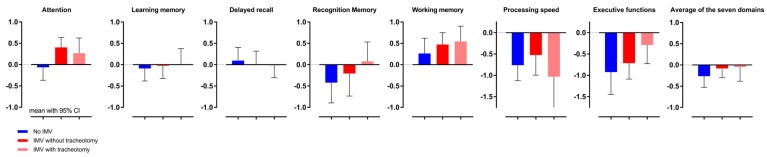
Bar graphs of domain z-scores, shown per ventilation group. The means of the z-scores are shown along with the 95% confident intervals. The values of individual domains differed: the mean values of Attention Index, Learning Memory Index, Delayed Recall Index, and Recognition Memory Index did not significantly differ from 0; the mean value of Working Memory Index was greater than 0; that of Processing speed index and of Executive Functions index were lower than zero. The average score over all patients was marginally lower than zero.

Of the total sample of 80 participants, the mean z-score for working memory was above zero, whereas the mean z-scores for processing speed and executive functions were below zero. The other domains did not differ from zero (see Figure 3).

Per person, the z-scores were averaged over the domains. The mean of these averaged z-scores was −0.138 (95% CI of the mean: −0.289 to 0.0128), which is marginally lower than zero; t(79) = 1.82, *p* = 0.072, Cohen’s *d* = 0.4.

Broken down into the three groups:

- Patients who did not receive IMV (*n* = 29) showed lower average z-scores than those who did receive IMV (*n* = 51), but this difference was not statistically significant: t(78) = 1.3, *p* = 0.21, Cohen’s *d* = 0.28.
- Patients with tracheostoma (*n* = 24) showed average z-scores comparable to those without tracheostoma (*n* = 27): t(49) = 0.18; *p* = 0.86, Cohen’s *d* = 0.05.

The *t* tests and Pearsons r’s of correlations between the domains are provided in Supplement S2.3.

### 3.3. Main Analyses: GlobCogPerf Versus IMV Days, Moderator Age

**Figure 4:**
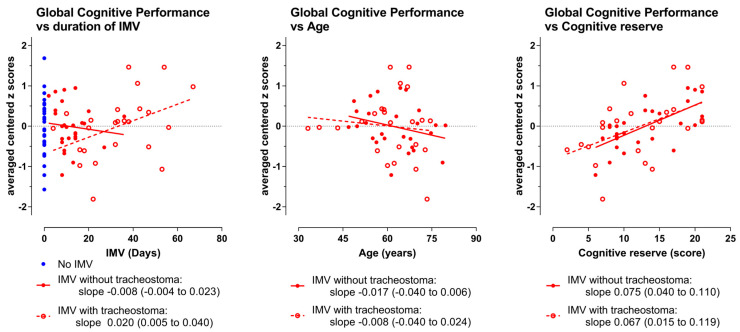
Scatter dot plots showing individual data points with corresponding partial regression lines. The individual averages of the per domain centered z-scores. The blue symbols represent the patients who were not IMV (*n* = 29); the closed red symbols represent the patients who received IMV without a tracheostoma (*n* = 27); and the open red symbols represent the patients who received IMV with a tracheostoma (*n* = 24). The lines drawn are the partial regression line of the averaged centered z-scores versus the independent variable indicated on the x-axis. Slope values with 95% CI are given.

Per ventilation group, we examined the association between the duration of mechanical ventilation (DoV) and GlobCogPerf, as well as its moderation by age. A linear regression model, Model 1, including only the main effect of DoV, was compared with Model 2, in which Age and the interaction term (DoV × Age) were added, to test for moderation. Summary statistical results of Model 1 (DoV) and Model 2 (Age and DoV × Age) are presented in Table 2. The complete statistical output is provided in Supplement S2.4. Scatter dot plots displaying individual GlobCogPerf scores, along with the corresponding partial regression lines are shown in Figure 4. For more detailed figures, see Supplement S2.5.

- *For the IMV group without a tracheostoma (n = 27):*

Model 1 (DoV) did not show a significant association between DoV and GlobCogPerf: R^2^ = 0.11; F(1,25) = 0.282; *p* = 0.6; Cohen *f*^2^ = 0.01. The coefficient for DoV was −0.01.

Model 2 (adding Age and DoV × Age), did not significantly improve the model fit compared with Model 1 (*p* = 0.39), effect size *f*^2^ = 0.10. Age showed a small negative association with cognitive functioning with a coefficient for Age of −0.016, and the interaction term was negligible, with a coefficient < 0.001.

- *For the IMV group with tracheostoma (n = 24):*

Model 1 (DoV) showed a significant positive association between DoV and GlobCogPerf: R^2^ = 0.17; F(1,22) = 4.514; *p* = 0.045; Cohen *f*^2^ = 0.21. The coefficient for DoV was 0.020.

Model 2 (adding age and DoV × Age) did not significantly improve the model fit compared with Model 1 (*p* = 0.42), effect size *f*^2^ = 0.32. Age showed a small negative association with cognitive functioning, with a coefficient for age of −0.01, and the interaction term was negligible, with a coefficient of 0.001.

### 3.4. Exploratory Analyses

#### 3.4.1. GlobCogPerf Versus IMV Days, Moderator Cognitive Reserve

We explored whether the association between the duration of mechanical ventilation (DoV) and GlobCogPerf was moderated by cognitive reserve (CogRes). The linear regression model Model 1 (DoV) was compared with Model 3, in which CogRes and the interaction term (DoV × CogRes) were added to test for moderation. Summary statistical results are presented in Table 2. The complete statistical output is provided in Supplement S2.4.

- *For the IMV group without a tracheostoma (n = 27):*

Model 1 (DoV), already given above, did not show a significant association between DoV and GlobCogPerf: *p* = 0.6, Cohen *f*^2^ = 0.01.

Model 3 (adding GogRes and DoV × CogRes) did significantly improve the model fit compared with the initial model (*p* < 0.001), with an effect size of *f*^2^ = 0.88. CogRes was the main factor for this fit improvement. The fit showed a large positive association of CogRes with GlobCogPerf, with a coefficient of 0.077, *p* < 0.001, while the interaction term was slightly negative with a coefficient of −0.002.

- *For the IMV group with tracheostoma (n = 24):*

Model 1 (DoV) already given above, showed a significant association between DoV and GlobCogPerf: *p* = 0.045; Cohen *f*^2^ = 0.21 and a coefficient for DoV of 0.020.

Model 3 (adding CogRes and DoV × CogRes) did significantly improve the model fit compared with the initial model (*p* = 0.037), and the effect size was *f*^2^ = 0.67. CogRes was the main factor for this fit improvement. The fit showed a large positive association of CogRes with GlobCogPerf with a coefficient of 0.067 (*p* = 0.01),while the interaction effect, with a coefficient of −0.001 (*p* = 0.94), was negligible.

Statistical results are presented in Table 2. The statistical output of SPSS is provided in Supplement S2.4. Scatter dot plots are shown in Figure 4. Detailed figures are provided in Supplement S2.5.

#### 3.4.2. CogPerf per Domain: IMV Days, Moderators Age, Cognitive Reserve

- *Regarding days of ventilation (DoV) per domain:*

In the IMV group without tracheostoma, the regression slopes relating CogPerf per domain to days of ventilation were not consistent in sign direction. The mean value of the slopes per domain did not differ significantly from zero.

In contrast, the slopes for the IMV group with a tracheostomy relating CogPerf per domain to DoV were all positive, with a mean value 0.023. This value is comparable to the fit value of GlobCogPerf to DoV, given above (i.e., 0.020). This finding supports the suggestion that, at least for the tracheostomized patients, longer duration of ventilation is associated with better cognitive outcomes.

- *Regarding age (Age) per domain:*

In none of the groups were the regression slopes relating CogPerf per domain to age consistent in direction, and none of the means of the slopes differed significantly from zero.

- *Regarding cognitive reserve (CogRes) per domain:*

In both ventilated groups, the regression slopes relating cognitive performance per domain to CogRes were all positive, with a mean value 0.075. This value is comparable to the fit value of the GlobCogPerf to CogRes given above (i.e., 0.075). This finding supports the suggestion that, in patients who underwent mechanical ventilation, higher cognitive reserve is associated with better cognitive outcomes following ICU discharge.

All figures, fit data and statistics of the domains are given in Supplement S2.6.

#### 3.4.3. Per Delirium: GlobCogPerf Versus IMV Days, Moderators Age, and Cognitive Reserve

In Figure 2, we show that patients with IMV experienced delirium significantly more often than those who did not receive IMV. The presence or absence of a tracheostomy did not influence this effect. However, the patients who experienced delirium had significantly longer DoV than those who did not (ANOVA main effect of delirium: F(1,47) = 5.55, *p* = 0.023). This effect was observed in both ventilation groups: the interaction between delirium and tracheostomy status was not significant (F(1,47) = 1.99, *p* = 0.17).

Among the patients with a tracheostomy, the association between GlobCogPerf and DoV was positive for both patients with and without delirium. The slopes, being 0.023 and 0.024, were comparable to that of the undivided group (i.e., 0.020). Moreover, between patients with and without delirium, GlobCogPerf, Age, and CogRes did not differ significantly (with all *t* tests *p*-values > 0.3). All figures, fit data, and statistics are given in Supplement S2.7.

## 4. Discussion

With this preregistered data analysis, we investigated the association between the duration of invasive mechanical ventilation (IMV) and cognitive functioning one year after ICU discharge, in a cohort of COVID-19 ICU survivors. In addition, we explored the moderating effects of age, cognitive reserve, and experience of delirium during ICU stay. We explored these relationships separately in patients who were ventilated without a tracheostoma and those ventilated with a tracheostoma. Among tracheostomized patients, longer IMV duration was associated with better cognitive outcomes. While age had a small negative impact, cognitive reserve showed a considerable positive association with cognitive outcome. Both the interaction terms had a negligible effect.

Cognitive functioning was, on average, slightly below norm scores. This finding reflects the results from the original paper, which reported that 24 of the 80 patients met the criteria for objective cognitive impairment [2]. Multiple studies report cognitive deficits in post-COVID-19 patients [27,28,29,30] which likely arise from a combination of contributing factors [31]. Cerebrovascular events, impaired cerebral perfusion, and in particular hypoxemia have been identified as key contributors [32,33]. Adingupu et al. [32] reported that the reduced oxygenation in the brain was indeed associated with reduced neurological function. Of course, hypoxemia is not unique to COVID-19, nor are the associated cognitive impairments. Cognitive impairment has also been documented among, for example, survivors of acute respiratory distress syndrome (ARDS) [34]. This highlights the brain’s vulnerability to hypoxic states and the relevance of optimal cerebral oxygenation in critical illness.

Oxygenation data were not available in our patient group; nevertheless, the comparison between patients who received IMV and those who did not receive IMV suggests that ventilation itself may be associated with better cognitive outcomes. This finding aligns with the study by Alemanno et al. [35], who found that COVID-19 patients requiring endotracheal intubation showed better cognitive recovery than those who either did not require respiratory assistance or managed with non-invasive ventilation. Although their intubated patients had more severe clinical courses, they had, surprisingly, a better cognitive outcome. This finding is also supported by the present data, showing that ventilated patients had higher APACHE II scores than those without IMV, yet their cognitive performance tended to be better.

The comparison between the IMV and non-IMV groups was, however, not the primary aim of our study. Our main objective was to examine the association between the *duration* of IMV and long-term cognitive outcomes. To address this question, we restricted our analyses to patients who had received IMV; these patients were further stratified according to whether or not they underwent tracheostomy.

In tracheostomized patients, longer mechanical ventilation duration was, unexpectedly, associated with better global cognition, whereas no such association appeared in non-tracheostomized patients. Interestingly, Rapin et al. [36], also in a COVID-19 cohort, identified the duration of oxygen therapy as the only predictor of cognitive outcome, with longer exposure showing a protective effect. Our results align with these results [36], as we also observed that longer ventilation duration was associated with better cognitive outcomes. And as reported by Alemanno in [35], this effect cannot be explained by differences in illness severity, since the two ventilated groups (with and without tracheostomy) did not differ in APACHE II scores. We suggest that sustained and optimal oxygen delivery during the acute phase of COVID-19 infection might protect against cognitive decline.

The finding that longer ventilation benefited only tracheostomized patients is remarkable and merits confirmation in studies with larger sample sizes (e.g., Hampshire et al., [27]). Although the ventilation duration of the tracheostomized patients exceeded that of intubated patients, which limits direct comparison, the result may guide hypothesis generation. We hypothesize that, in tracheostomized patients, not only may the length of ventilation contribute positively, but tracheostomy itself may also provide more efficient respiratory support. To our knowledge, no studies have directly compared physiological indices between endotracheally intubated ICU patients and those with tracheostomies, though the findings of Lin et al. [37], which show lower ICU and in-hospital mortality among tracheostomized patients, provide some support for this hypothesis.

Other factors might also influence cognitive outcomes, such as the fact that tracheostomy reduces the use of sedative and analgesic medications [38,39] compared with endotracheal intubation. IMV with endotracheal intubation typically requires prolonged sedation, which is linked to adverse cognitive outcomes in ICU survivors [40]. Although data on sedative prescriptions were unavailable in our cohort, sedation may have contributed to the cognitive outcomes in our study population [41].

Sedation is also a risk factor for developing delirium [42], and delirium has been associated with long-term cognitive decline [40,42,43,44]. In our cohort, delirium was observed among ventilated patients, with no significant difference between the tracheostomy and intubation-only groups. The positive association between longer ventilation duration and better cognitive outcomes was seen in both delirium and non-delirium patients with tracheostomy. However, some notes on this point. First, the diagnosis of delirium should be interpreted cautiously [45], as delirium was assessed retrospectively from medical records during a period of extremely high clinical workload of the medical personnel, making underreporting and inconsistent documentation likely. Second, the type of sedative is likely an important factor in long-term cognitive impairment, with benzodiazepines being a particular risk factor [9]. We lack data on the sedatives administered; however, it is plausible that the overwhelming ICU workload, much like the challenges in diagnosing delirium, shifted focus away from preventing long-term outcomes and optimizing sedation strategies [40]. Moreover, Nedergaard et al. [14] emphasize that the relationship between sedation, delirium, and cognitive outcomes is not straightforward. Taken together, the heterogenicity and complexity of ICU patients and the challenging conditions in the ICU make it difficult to isolate the effects of a single factor. Therefore, it remains an open question to what extent the pharmacological effects of sedatives, sedation, and delirium are associated with the observed cognitive deficits.

Age frequently emerges as a risk factor for cognitive difficulties among ICU survivors [46,47,48], but in our cohort we observed only a slight negative relationship with age. Age also did not significantly modify the positive association between longer ventilation duration and GlobCogPerf.

We did, however, observe a significant positive relationship between GlobCogPerf and cognitive reserve. This positive association of cognitive reserve with post ICU cognitive outcomes has also been well documented [25,26,48]. Recent analyses have shown that higher levels of cognitive reserve are associated with less cognitive impairment, even among individuals with similar clinical vulnerability [49]. This finding is supported by the present data: although ventilated patients had higher APACHE II scores than those not receiving IMV, their cognitive performance tended to be better. Neither age nor cognitive reserve modified the association between longer ventilation duration and global cognitive performance. Therefore, our data suggest that ventilation, age, and cognitive reserve are independent factors. While age and cognitive reserve are non-modifiable once patients are in the ICU, the mode and duration of ventilation are modifiable factors.

Our central finding is that, among tracheostomized patients, longer IMV is associated with higher GlobCogPerf. Although it is tempting to attribute this positive association to a protective effect of prolonged IMV, causal inference cannot be drawn from this dataset. The observed association may be driven by clinical decision-making bias, unmeasured confounders (e.g., premorbid frailty, socioeconomic factors, and access to rehabilitation), or even by reverse causation. Therefore, the finding should be regarded as hypothesis-generating and suggests that sustained and optimized ventilatory support during the acute phase of illness *may* be relevant to the preservation of long-term brain health in COVID-19 and other hypoxia-related conditions.

Ventilation extending beyond the acute period may also be advisable (e.g., via home oxygen devices), as Adingupu et al. [32] demonstrated that hypoxia can persist for months after ICU discharge, even in asymptomatic individuals with post-acute COVID-19. This underscores the need to maintain adequate respiratory support beyond the acute phase.

## 5. Conclusions

In summary, tracheostomy-based ventilation may offer advantages compared with endotracheal intubation, including potentially improved oxygen delivery and reduced need for sedative medications. However, ventilation via tracheostomy, as with endotracheal intubation, involves positive pressure ventilation (PPV), which delivers supra-atmospheric pressure through the airways. PPV includes the risk of developing ventilator-induced lung injury by raising the intrathoracic pressure [5,50] and of causing subsequent harmful neurosignalling [4,5,15]. Elevated intrathoracic pressure also impedes venous return and cardiac output, compromising cardiovascular performance [18,51]. These limitations highlight the need for safer alternatives for long-term ventilatory support.

In this context, a nearly forgotten but physiologically favorable mode of ventilation warrants renewed attention: negative pressure ventilation (NPV) [52,53,54]. By applying sub-atmospheric pressure around the torso, NPV supports natural respiratory mechanics without elevating intrathoracic pressure [50,51]. Consequently, NPV not only minimizes lung injury but also enhances cardiovascular, and thus cerebral perfusion [51,55]. Therefore, our findings, combined with concerns about PPV-related VABI, indicate that alternative modes such as NPV warrant evaluation in future trials.

## Figures and Tables

**Table 1:** The variables present in the obtained raw data that are used in the present analysis.

Independent Variables	Dependent Variables
Invasive Mechanical Ventilation (days)	Attention Index
Tracheostomy (yes/no)	Learning Memory Index
Age (years)	Delayed Recall Index
Cognitive Reserve (score)	Recognition Memory Index
CCI comorbidity (score)	Working Memory Index
APACHE II (score)	Processing speed Index
Delirium (yes/no)	Executive Functions Index

**Table 2:** Summary of linear regression results for GlobCogPerf versus DoV, with moderation by age and CogRes. Full results are provided in Supplement S2.4.

Model		R^2^	Adjusted R^2^	Sig. F Change *p*	Cohen’s *f*^2^	Coeff. a DoV	*p*	Coeff. b Mod.	*p*	Coeff. c Interact.	*p*
	**Without tracheostoma**										
1	DoV only	0.011	−0.280	*0.600*	0.011	−0.008	*0.600*				
2	DoV, Age, (DoV × Age)	0.090	−0.290	*0.385*	0.099	−0.004	*0.788*	−0.160	*0.186*	0.001	*0.773*
3	DoV, CogRes, (DoV × GogRes)	0.467	0.397	*0.001*	0.875	−0.005	*0.743*	0.077	*0.0002*	−0.002	*0.509*
	**With tracheostoma**										
1	DoV only	0.170	0.133	*0.045*	0.205	0.020	*0.045*				
2	DoV, Age,(DoV × Age)	0.240	0.125	*0.418*	0.315	0.025	*0.045*	−0.009	*0.545*	0.001	*0.328*
3	DoV, CogRes, (DoV × GogRes)	0.403	0.313	*0.037*	0.674	0.016	*0.088*	0.067	*0.011*	<0.001	*0.939*

## Data Availability

Data are supplied in Supplement S1.

## Supplementary Materials

The following supporting information can be downloaded at https://www.mdpi.com/article/10.3390/jcm15020898/s1. Supplement S1: Raw data used in this study; Supplement S2: S1: Figure and table depicting correlations among duration of DoV, age, and CogRes; S2: Figure and table illustrating correlations between DoV, CCI, and APACHE II; S3: Graphs of mean z-scores of the seven cognitive domains with t-tests and table illustrating correlations between the scores of the seven domains; S4: SPSS output multiple linear regressions; S5: GloCogPef detailed partial linear regression graphs and table with statistics; S6: Per domain graphs with linear regression lines and tables with statistics; S7: Graphs per delirium group.

## Abbreviations

The following abbreviations are used in this manuscript:

APACHE II	Acute Physiology and Chronic Health Evaluation II
ARDS	acute respiratory distress syndrome
CCI	Charlson Comorbidity Index
CRQ	Cognitive Reserve Questionnaire
CogPerf	cognitive performance
CogRes	cognitive reserve
DoV	days or duration of ventilation
GlobCogPerf	global cognitive performance
ICU	intensive care unit
IMV	invasive mechanical ventilation
NPV	negative pressure ventilation
PPV	positive pressure ventilation
VABI	ventilator-associated brain injury
