# Supplementary material for: Rethinking Mechanical Ventilation: Can Ventilation Mode Influence Long-Term Cognitive Outcomes in ICU Patients with COVID-19?
Source: J Clin Med. 2026 Jan 22;15(2):898. doi: 10.3390/jcm15020898 (PMC12842449; doi:10.3390/jcm15020898)
Supplement: Supplementary file 1 [file jcm-15-00898-s001.zip › S1_Rethinking Mechanical Ventilation_VanRijn et al_raw data.pdf]

Data used in the paper: **Rethinking Mechanical Ventilation: Can Ventilation Mode Influence Long-Term Cognitive Outcomes in ICU Patients? by Van Rijn et al,.**

It concerns a secondary analysis using data from a study of Covid-19 ICU survivors, published by Godoy-González et al.
Reference:

Godoy-González M, Navarra-Ventura G, Gomà G, de Haro C, Espinal C, Fortià C, Ridao N, Miguel Rebanal N, Oliveras-Furriols L, Subirà C, Jodar M, Santos-Pulpón V, Sarlabous L, Fernández R, Ochagavía A, Blanch L, Roca O, López-Aguilar J, Fernández-Gonzalo S.

Objective and subjective cognition in survivors of COVID-19 one year after ICU discharge: the role of demographic, clinical, and emotional factors.
Crit Care. 2023;27(1):188. Published 2023 May 15. doi:10.1186/s13054-023-04478-7
https://doi.org/10.1186/s13054-023-04478-7

**Independent variabels**	**Comments**
Age	years
Sex (0/1)	0=male ; 1=female
Delirium	Presence of delirium: 0= No; 1= Yes
CCI	Charlson Comorbidity Index
APACHE II	Score of Acute Physiology and Chronic Health Evaluation
Mechanical Ventilation (days)	Lenght of Mechanical Ventilation (days of invasive ventilation)
Tracheostomy	0= No; 1= Yes; 2= NA (Not applicable)
Cognitive reserve	Cognitive Reserve Questionnaire, raw score (0-25)

**Dependent variabels**	
Attention index: n=80	(Digit span forward WAIS-III+ Spatial score forward WMS-III)/2)
Learning Memory index: n=80	(Number of words learned RAVLT + Number of tokens learned SPART)/2)
Delayed Recall index: n=80	(Nº of words recalled in the long term RAVLT + Number of tokens recalled in the long term SPART)/2)
Recognition Memory index: n=80	(Memory Recognition, Nº of words RAVLT recognized in the long term)
Working Memory index: n=80	(Working Memory (Digit span backwards WAIS-III + Spatial score backwards WMS-III)/2"
Processing speed index: n=77	(Processing speed (TMT A + Number of words read in Stroop test)/2)
Executive Functions index: n=80	(TMT B - TMT A + Interference score Stroop Test + phonetic verbal fluency, FAS)/3)

These indexes were obtained from the orinal authors. They calaculted them from objective cognitive performance tests.
These tests are listed in the original publication by Godoy-González et al., DOI: 10.1186/s13054-023-04478-7

 Data used in the paper: **Rethinking Mechanical Ventilation: Can Ventilation Mode Influence Long-Term Cognitive Outcomes in ICU Patients? by Van Rijn et al,.**

ID	Age (years)	Sex (0/1)	Delirium (0/1)	CCI	APACHE II	Mechanical Ventilation (Days)	Tracheostomy (0/1/2)	Cognitive Reserve z-scores	Attention index z-scores	Learning Memory index z-scores	Delayed Recall index z-scores	Recognition Memory index z-scores	Working Memory index z-scores	Processing speed index z-scores	Executive Functions index z-scores
**No Invasive Mechanical Ventilation**															
1	71.05	1	1	2	17	0	2	9	0.23	-0.03	-0.29	-2.80	0.48	-0.55	-0.57
2	48.10	0	0	0	8	0	2	18	0.15	-0.99	-0.16	0.69	0.34	0.64	0.02
3	67.80	0	0	2	9	0	2	4	-0.42	0.31	1.28	-0.11	-0.78	-0.84	-2.07
4	50.75	1	0	0	6	0	2	15	-0.18	0.51	1.07	0.70	0.01	-0.47	-0.61
5	48.93	0	0	0	5	0	2	13	-0.18	0.75	1.21	-2.00	-0.37	0.19	-0.74
6	56.81	1	0	3	18	0	2	11	-0.02	-0.40	0.24	0.96	-0.65	-0.51	0.00
7	49.33	0	1	1	5	0	2	14	-0.20	-0.61	-0.51	0.31	-1.09	-1.12	-3.53
8	42.55	1	0	0	2	0	2	8	-0.88	0.93	0.43	0.91	-0.37	0.03	-0.74
9	78.75	1	0	3	12	0	2	9	0.49	0.64	0.89	-0.80	1.51		1.05
10	43.00	0	0	1	3	0	2	18	0.17	-0.28	-0.15	0.69	0.78	0.08	-0.14
11	47.42	0	0	0	7	0	2	12	-1.70	-1.11	-0.70	-2.38	-1.09	-2.07	-3.83
12	46.77	0	0	3	8	0	2	11	0.17	0.35	0.49	-0.46	1.01	0.19	-0.67
13	69.83	0	0	3	10	0	2	6	-0.07	-0.49	-0.36	-0.64	0.49	-2.45	-1.14
14	58.41	0	0	1	9	0	2	7	-0.92	-0.75	-0.43	-0.52	0.03	-1.36	-0.58
15	57.93	0	0	1	5	0	2	14	0.39	-0.17	0.05	-0.15	0.15	-0.87	-0.04
16	70.32	0	0	3	8	0	2	6	0.23	0.75	1.00	-0.19	1.35	-0.77	0.31
17	60.48	0	0	3	8	0	2	9	-0.92	-0.15	-0.47	-0.33	-0.65	-3.27	-4.61
18	39.48	1	0	0	2	0	2	12	-1.70	-1.65	-0.44	0.21	-1.47	-0.43	-1.55
19	70.92	1	0	3	12	0	2	9	1.09	-1.45	-1.99	-3.30	1.01	-2.86	-1.34
20	37.08	0	0	1	10	0	0	12	-0.68	-0.89	-0.81	-1.37	0.13	-0.62	-0.76
21	67.63	0	0	3	6	0	2	8	0.39	1.17	0.99	0.27	0.65	-0.27	-1.34
22	79.07	0	0	3	7	0	0	6	-0.44	-0.86	-0.96	-0.96	0.32	-0.65	-0.05
23	48.80	0	0	0	3	0	2	12	0.67	0.54	1.05	1.08	1.44	-0.11	0.35
24	55.60	0	0	1	2	0	0	10	-0.45	-0.26	0.47	0.22	-0.31	-1.26	-1.84
25	73.06	1	0	3	7	0	0	5	-0.01	-0.60	-0.77	-3.30	0.52	-0.05	-0.96
26	60.22	0	0	2	3	0	0	9	0.65	-0.07	-0.35	0.27	0.19	-0.69	-2.44
27	67.07	0	0	3	8	0	0	9	0.32	0.45	-0.08	-0.03	1.15	0.19	0.06
28	69.87	0	0	2	5	0	0	11	2.35	1.25	1.23	0.58	3.00	-0.19	1.68
29	60.02	0	0	1	5	0	0	11	-0.35	0.59	0.86	0.27	-0.15	-1.23	-0.70

pag3/4 Data used in the paper: **Rethinking Mechanical Ventilation: Can Ventilation Mode Influence Long-Term Cognitive Outcomes in ICU Patients? by Van Rijn et al,.**

ID	Age (years)	Sex (0/1)	Delirium (0/1)	CCI	APACHE II	Mechanical Ventilation (Days)	Tracheostomy (0/1/2)	Cognitive Reserve z-scores	Attention index z-scores	Learning Memory index z-scores	Delayed Recall index z-scores	Recognition Memory index z-scores	Working Memory index z-scores	Processing speed index z-scores	Executive Functions index z-scores
**Invasive Mechanical Ventilation without tracheostoma**															
30	68.36	1	1	3	9	27	0	9	-0.42	-1.56	-1.11	0.61	-0.15	-1.59	0.02
31	70.69	0	0	3	8	5	0	13	0.92	1.81	0.45	-0.19	0.68	-0.19	-1.23
32	56.72	1	0	1	4	5	0	21	0.45	1.51	1.36	1.07	0.65	0.18	0.28
33	53.74	0	1	1	5	5	0	15	0.17	0.16	0.67	0.96	-0.37	0.29	-0.19
34	49.75	0	1	0	8	8	0	9	0.37	0.17	-0.41	-0.85	0.56	-0.17	-0.17
35	48.73	0	0	0	3	8	0	19	0.70	0.21	-0.31	-0.08	1.82	0.79	0.72
36	61.30	1	1	2	8	8	0	6	-0.85	-0.86	0.16	0.58	-0.65	-5.00	-2.37
37	67.82	1	0	3	9	8	0	14	0.39	-0.89	-1.14	-0.82	0.16	-0.31	-0.08
38	56.31	1	1	2	8	8	0	12	-0.42	-0.75	-0.03	-0.41	0.15	-1.59	-0.27
39	68.87	0	1	2	8	9	0	17	-0.18	-1.00	-0.97	-0.03	0.15	-0.26	-2.44
40	66.29	1	1	2	11	9	0	20	1.52	0.81	0.05	1.32	1.95	-0.51	0.68
41	67.17	0	1	5	20	9	0	10	0.22	-0.26	-0.38	-0.94	-0.61	-2.07	-1.19
42	76.33	1	0	3	21	9	0	20	0.32	0.23	-0.68	-1.80	1.38	0.01	0.22
43	46.90	0	0	0	7	10	0	8	0.51	-0.06	0.97	-1.62	0.30	0.39	-1.15
44	55.07	0	1	1	8	11	0	7	-0.42	-0.36	-0.20	0.59	0.03	-1.33	-0.92
45	78.65	0	0	5	14	13	0	8	0.92	-0.25	-0.29	-4.42	0.32	-1.55	-1.55
46	65.49	1	1	2	18	14	0	9	-0.35	0.53	-0.15	0.96	0.15	-1.08	-2.40
47	64.36	0	1	4	6	14	0	19	0.55	1.15	1.42	1.48	0.78	-0.08	0.82
48	74.74	1	1	4	7	14	0	10	-0.01	0.40	0.14	-1.80	-0.02	0.25	-0.65
49	58.11	0	0	2	14	14	0	9	0.95	0.00	0.70	-0.89	0.15	-0.70	-2.08
50	59.05	1	1	3	10	14	0	9	0.15	-0.69	-1.39	0.33	-0.15	0.10	-0.99
51	52.79	0	0	1	9	17	0	10	1.02	-0.13	-0.20	-0.52	0.34	0.34	-0.80
52	70.15	0	0	5	6	18	0	18	0.89	0.12	-0.03	-2.12	1.62	0.34	-0.84
53	79.64	0	0	4	12	18	0	8	1.35	-0.46	-0.58	0.19	1.38	-1.16	-1.09
54	49.59	0	1	0	9	20	0	14	0.16	-0.30	0.46	0.69	0.13	0.69	0.26
55	63.01	0	1	2	10	36	0	21	0.95	-0.37	0.14	1.18	0.78	-0.52	-0.96
56	54.25	0	0	1	5	2		13	1.02	0.22	1.63	0.96	1.28	0.59	-0.92

 Data used in the paper: **Rethinking Mechanical Ventilation: Can Ventilation Mode Influence Long-Term Cognitive Outcomes in ICU Patients? by Van Rijn et al,.**

ID	Age (years)	Sex (0/1)	Delirium (0/1)	CCI	APACHE II	Mechanical Ventilation (Days)	Tracheostomy (0/1/2)	Cognitive Reserve z-scores	Attention index z-scores	Learning Memory index z-scores	Delayed Recall index z-scores	Recognition Memory index z-scores	Working Memory index z-scores	Processing speed index z-scores	Executive Functions index z-scores
**Invasive Mechanical Ventilation with tracheostoma**															
57	33.15	1	0	0	4	4	1	19	-0.33	-0.52	0.52	0.21	-0.36	-0.17	-0.06
58	55.69	1	0	1	5	10	1	11	0.15	-0.18	0.16	-0.04	0.75		0.74
59	71.72	0	1	4	32	21	1	21	1.09	-0.39	-0.20	0.58	1.36	-1.65	-0.09
60	72.76	0	1	3	7	16	1	2	-0.28	-0.44	-0.21	-1.35	-0.89	-0.15	-1.11
61	60.05	1	1	2	8	16	1	6	-0.42	-0.35	-0.47	-0.46	0.03	-3.38	-2.12
62	56.63	0	0	1	13	18	1	12	-0.35	-0.47	-0.67	-0.89	-0.31	-1.40	-0.52
63	43.22	0	1	0	3	20	1	14	-0.35	-0.18	0.39	0.69	-0.37	-0.13	-0.69
64	73.42	1	0	3	12	22	1	7	-0.51	-1.31	-0.87	-2.80	0.68	-7.64	-0.55
65	62.32	1	0	2	18	23	1	13	-0.25	-1.79	-1.38	0.25	0.15	-2.43	-1.34
66	69.62	0	1	3	10	32	1	4	-0.08	-0.78	-0.50	0.88	-0.31	-2.25	-0.49
67	61.05	0	1	2	3	32	1	7	-0.03	0.45	-0.52	-0.94	0.31		0.96
68	58.85	0	0	3	14	33	1	17	1.24	-0.41	0.02	0.22	1.47	0.38	-0.38
69	51.38	0	1	1	4	33	1	15	-0.35	0.73	0.37	1.33	0.13	-0.47	-1.26
70	74.52	1	1	4	24	36	1	9	0.99	0.12	-0.51	-1.30	1.85	-0.54	-0.03
71	67.35	0	1	4	10	38	1	17	1.12	0.05	-0.77	-0.03	0.65	-0.80	0.22
72	68.46	0	1	2	9	38	1	21	1.95	1.75	0.72	0.58	2.08	1.41	1.42
73	64.39	0	0	2	14	42	1	10	1.55	1.15	0.08	1.18	2.08	0.10	0.96
74	58.13	0	1	2	8	43	1	8	0.29	0.43	0.70	0.96	0.49	-0.27	0.07
75	63.96	1	1	2	12	47	1	5	-1.15	-0.56	-0.18	-0.46	-0.31	-0.50	-0.76
76	59.01	0	0	1	13	47	1	16	-0.25	0.22	0.93	0.96	0.49	-0.22	-0.06
77	69.65	0	1	3	12	53	1	14	-0.32	-0.57	-0.29	-1.24	0.19	-2.73	-2.86
78	60.98	0	1	1	11	54	1	19	2.02	2.23	1.72	1.48	1.62	-0.62	1.44
79	36.97	0	1	0	15	56	1	7	0.32	-0.07	-0.18	0.59	0.13	-0.47	-0.87
80	66.80	0	1	2	11	67	1	21	0.39	0.87	0.78	1.48	1.25	1.24	0.49
