# Supplementary material for: Rethinking Mechanical Ventilation: Can Ventilation Mode Influence Long-Term Cognitive Outcomes in ICU Patients with COVID-19?
Source: J Clin Med. 2026 Jan 22;15(2):898. doi: 10.3390/jcm15020898 (PMC12842449; doi:10.3390/jcm15020898)

Rethinking Mechanical Ventilation: Can Ventilation Mode Influence Long-Term Cognitive Outcomes in ICU Patients with COVID-19?

Clementina M. van Rijn [1,2,*], Marta Godoy-González [3,4], Sol Fernández-Gonzalo [3,5], Pierre Souren [6], Malcolm G. Coulthard [2,7], David J. Howard [2,8] and Marijtje L. A. Jongsma [9,10]

1 Donders Institute for Brain, Cognition and Behaviour, Radboud University, 6525 GD Nijmegen, The Netherlands tineke.vanrijn@donders.ru.nl
2 The Exovent Development Group, Wraysbury, TW19 5JF, UK; malcolm.coulthard@nhs.net (M.G.C.); davidjhoward10@gmail.com (D.J.H.)
3 Critical Care Department, Hospital Universitari Parc Taulí, Universitat Autònoma de Barcelona, Institut d'Investigació i Innovació Parc Taulí (I3PT-CERCA), 08208 Sabadell, Spain; mrgodoy@tauli.cat (M.G.-G.); msfernandez@tauli.cat (S.F.-G.)
4 Centro de Investigación Biomédica en Red de Enfermedades Respiratorias (CIBERES), Instituto de Salud Carlos III, 28029 Madrid, Spain
5 Centro de Investigación Biomédica en Red de Salud Mental (CIBERSAM), Instituto de Salud Carlos III, 28029 Madrid, Spain
6 Research Technical Support Group, Department of Psychology, Radboud University, 6525 GD Nijmegen, The Netherlands; steen.souren@ru.nl
7 Translational and Clinical Research Institute, Newcastle University, Newcastle upon Tyne NE1 7RU, UK
8 Imperial and UCLH Trust Hospitals, NW1 2BU, London, UK
9 Behavioural Science Institute, Radboud University, 6500 HE Nijmegen, The Netherlands; marijtje.jongsma@ru.nl
10 Faculty of Psychology, Universitas Gadjah Mada, Yogyakarta 55281, Indonesia
* Correspondence: tineke.vanrijn@donders.ru.nl

Supplement 2

S1 Correlations between DoV, Age, CogRes 2
Figure S1 Correlations duration of IMV (days), Age (years), Cognitive Reserve (CogRes)(score) 2
Table S1 Correlations duration of IMV (days), Age (years), Cognitive Reserve (CogRes)(score) 2
S2 Correlations between DoV, CCI, APACHE 3
Figure S2 Correlations between duration of Invasive Mechanical Ventilation (IMV)(days), CCI, APACHE 3
Table S2 Correlations between duration of Invasive Mechanical Ventilation (IMV)(days), CCI, APACHE 3
S3 The scores of the seven cognitive domains 4
Figure S3 T-tests 4
Table S3 Correlations between the scores of the seven cognitive domains 4
S4 SPSS output Multiple Linear Regression, with and without tracheostoma 5
Variables used 5
S4.1. Regression GlobCogPerf versus DoV; modulators Age and (DoV * Age) 5
S4.2 Regression GlobCogPerf versus DoV; modulators Age and (DoV * Age) 6
S4.3 Regression GlobCogPerf versus DoV; modulators CogRes and (DoV * Cogres) 7
S4.4 Regression GlobCogPerf versus DoV; modulators CogRes and (DoV * CogRes) 8
S5 Detailed partial linear regression graphs 9
Figure S5 Averaged centered z scores versus each of the three independent variables 9
Table S5 Outcomes Partial linear regression outcomes 9
S6 Per domain Linear Regression, with and without tracheostoma 10
Figure S6 Per domain Linear Regression, with and without tracheostoma 10
Table S6 Per domain Slopes of Linear Regression, with and without tracheostoma 11
S7 Delirium 12
Figure S7.1 For patients with and without delirium DoV, Age, CogRes and GlobCogPerf. 12
Figure S7.2 For patients with and without delirium: linear regression of GlobCogPerf vs. IMV days 12

S1 Correlations between DoV, Age, CogRes

Figure S1 Correlations duration of IMV (days), Age (years), Cognitive Reserve (CogRes)(score)

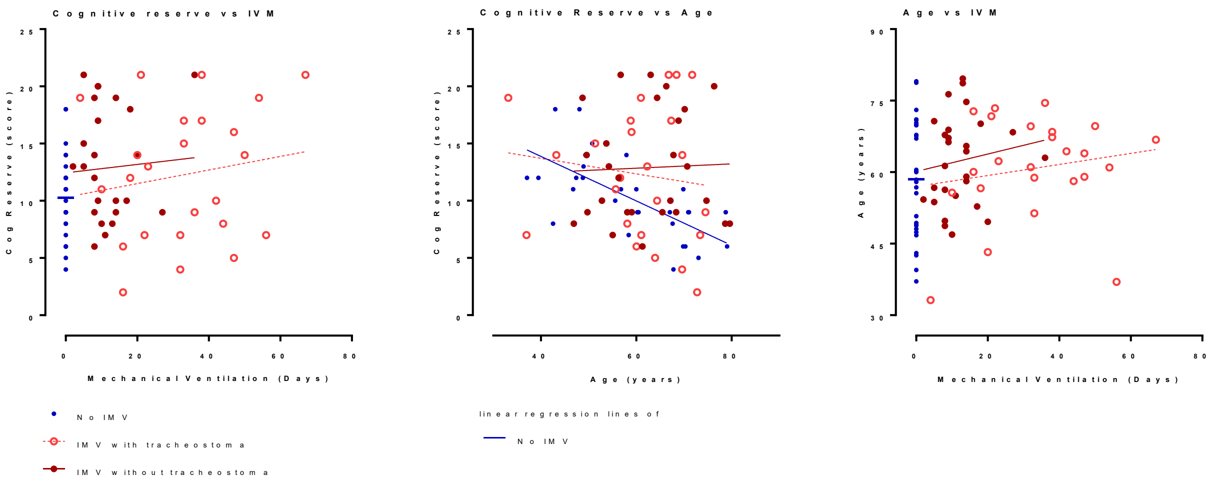

Table S1 Correlations duration of IMV (days), Age (years), Cognitive Reserve (CogRes)(score)

	**IMV (Days) vs. CogRes IMV with tracheostoma**	**IMV (Days) vs. CogRes IMV without tracheostoma**
Pearson r	0.1619	0.05396
95% confidence interval	-0.2584 to 0.5306	-0.3329 to 0.4253
R squared	0.02621	0.002912
P (two-tailed)	0.4497	0.7892
Significant? (alpha = 0.05)	No	No
Number of XY Pairs	24	27

	**IMV (Days) vs. Age (years) IMV with tracheostoma**	**IMV (Days) vs. Age (years) IMV without tracheostoma**
Pearson r	0.1691	0.1373
95% confidence interval	-0.2514 to 0.536	-0.2561 to 0.4916
R squared	0.02861	0.01884
P (two-tailed)	0.4295	0.4948
Significant? (alpha = 0.05)	No	No
Number of XY Pairs	24	27

	**Age vs. CogRes No IMV**	**Age vs. CogRes IMV with tracheostoma**	**Age vs. CogRes IMV without tracheostoma**
Pearson r	-0.6657	-0.1275	0.03681
95% confidence interval	-0.8298 to -0.3958	-0.5049 to 0.2909	-0.3481 to 0.4111
R squared	0.4432	0.01625	0.001355
P (two-tailed)	<0.0001	0.5528	0.8554
Significant? (alpha = 0.05)	Yes	No	No
Number of XY Pairs	29	24	27

S2 Correlations between DoV, CCI, APACHE

Figure S2 Correlations between duration of Invasive Mechanical Ventilation (IMV)(days), CCI, APACHE

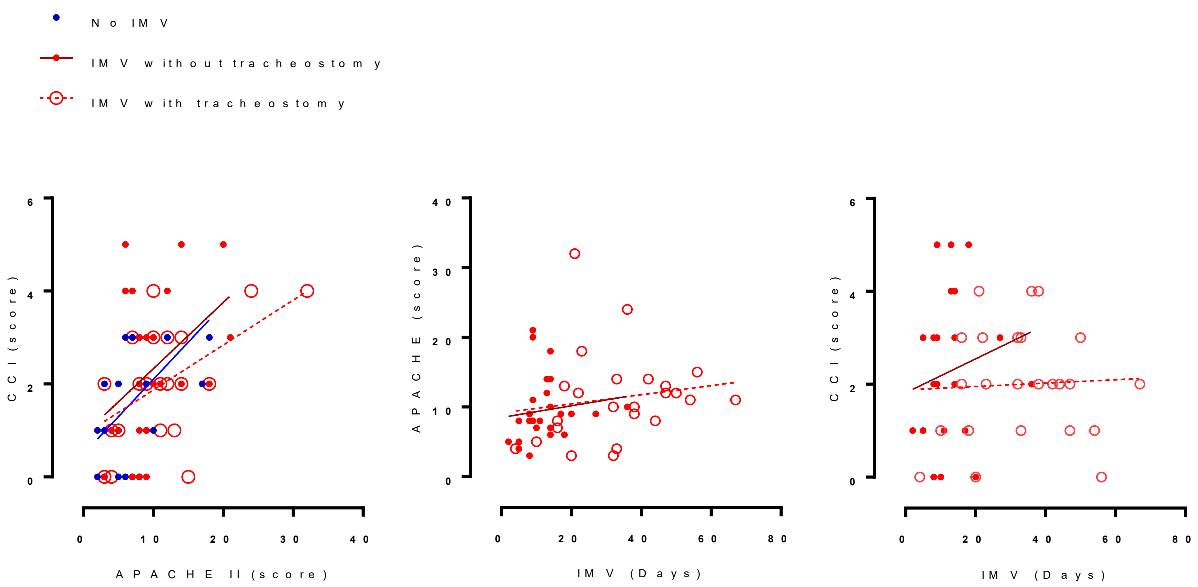

Table S2 Correlations between duration of Invasive Mechanical Ventilation (IMV)(days), CCI, APACHE

	APACHE II vs. CCI<br>No IMV	APACHE II vs. CCI<br>IMV with tracheostoma	APACHE II vs. CCI<br>IMV without tracheostoma
Spearman r	0.5641	0.3553	0.3931
95% confidence interval	0.2384 to 0.7757	-0.06874 to 0.6706	0.003559 to 0.6791
P (two-tailed)	0.0014	0.0884	0.0425
Exact or approximate P value?	Approximate	Approximate	Approximate
Significant? (alpha = 0.05)	Yes	No	Yes
Number of XY Pairs	29	24	27

	IMV (Days) vs. APACHE II<br>IMV with Tracheostoma	IMV (Days) vs. APACHE II<br>IMV without Tracheostoma
Spearman r	0.3527	0.3955
95% confidence interval	-0.07164 to 0.669	0.006439 to 0.6806
P (two-tailed)	0.0909	0.0411
Exact or approximate P value?	Approximate	Approximate
Significant? (alpha = 0.05)	No	Yes
Number of XY Pairs	24	27

	IMV (Days) vs. CCI<br>IMV with Tracheostoma	IMV (Days) vs.CCI<br>IMV without Tracheostoma
Spearman r	0.02914	0.2767
95% confidence interval	-0.3895 to 0.4378	-0.1271 to 0.6018
P (two-tailed)	0.8925	0.1624
Exact or approximate P value?	Approximate	Approximate
Significant? (alpha = 0.05)	No	No
Number of XY Pairs	24	27

S3 The scores of the seven cognitive domains

Figure S3 T-tests

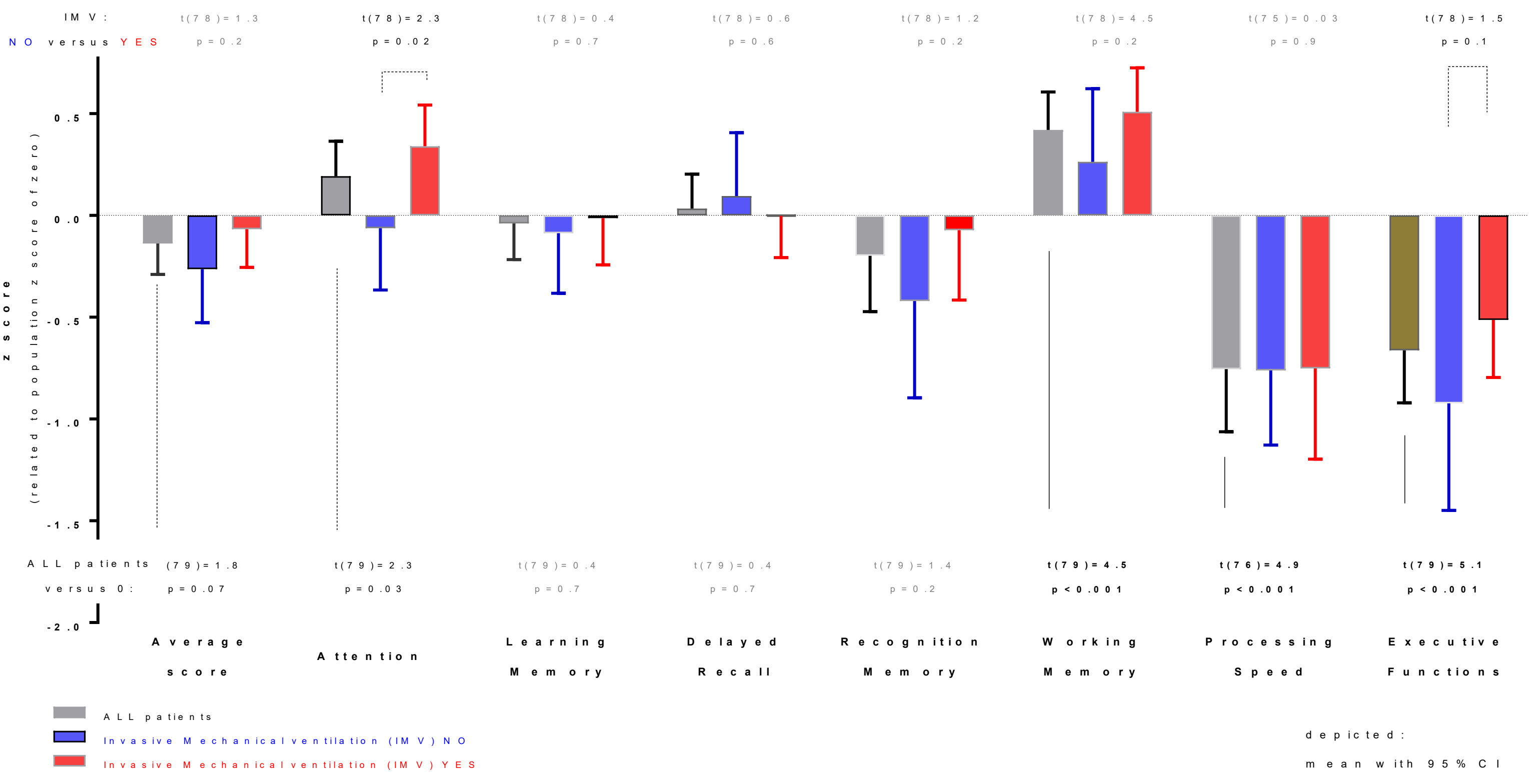

Table S3 Correlations between the scores of the seven cognitive domains

To examine the validity of averaging the 7 domains into one averaged score we determined the correlation between the cognitive performances.

Pearson r	averaged Zscore	Attention	Learning Memory	Delayed Recall	Recog Memory	Work Memory	Proces Speed	Executive Functions
averagedZscore		0.69	0.78	0.66	0.56	0.68	0.71	0.75
Attention	0.69		0.52	0.25	0.13	0.81	0.35	0.51
LearningMemory	0.78	0.52		0.74	0.33	0.49	0.46	0.42
DelayedRecall	0.66	0.25	0.74		0.43	0.21	0.39	0.30
RecogMemory	0.56	0.13	0.33	0.43		0.10	0.28	0.26
WorkMemory	0.68	0.81	0.49	0.21	0.10		0.27	0.62
ProcessSpeed	0.71	0.35	0.46	0.39	0.28	0.27		0.44
ExecutiveFunctions	0.75	0.51	0.42	0.30	0.26	0.62	0.44	

Pearson r (n= 80, except for ProcessSpeed: n=77)

Overall, the domain scores were highly correlated; however, their z-values differed (see Figure S3)
To investigate the influence of ventilation duration, we first accounted for differences in mean values across domains by centering each domain around its mean. For each patient, the seven centered domain scores were then averaged to obtain an individual global cognitive performance score (GlobCogPerf). Subsequently, the individual averaged centered cognitive scores of the 51 patients who received IMV were used in the pre-registered multiple linear regression analyses.

S4 SPSS output Multiple Linear Regression, with and without tracheostoma

Variables used

Dependent:

GlobCogPerf: Global Cognitive performance. (averaged centered domain z scores)

Independents:

DoV: Duration of ventilation (days)

Age (years)

GogRes: Cognitive reserve (score)

S4.1. Regression GlobCogPerf versus DoV; modulators Age and (DoV * Age)

Patients without tracheostoma, n=27.

Variables Entered/Removed[a]

Model	Variables Entered	Variables Removed	Method
1	DoV[b]	.	Enter
2	DoV_times_Age, Age[b]	.	Enter

a. Dependent Variable: GlobCogPerf
b. All requested variables entered.

Model Summary

Model	R	R Square	Adjusted R Square	Std. Error of the Estimate	Change Statistics: R Square Change	F Change	df1	df2	Sig. F Change
1	.106[a]	.011	-.028	.5563103	.011	.282	1	25	.600
2	.300[b]	.090	-.029	.5564283	.079	.995	2	23	.385

a. Predictors: (Constant), DoV
b. Predictors: (Constant), DoV, Age, DoV_times_Age

ANOVA[a]

Model		Sum of Squares	df	Mean Square	F	Sig.
1	Regression	.087	1	.087	.282	.600[b]
	Residual	7.737	25	.309		
	Total	7.824	26			
2	Regression	.703	3	.234	.757	.530[c]
	Residual	7.121	23	.310		
	Total	7.824	26			

a. Dependent Variable: GlobCogPerf
b. Predictors: (Constant), DoV
c. Predictors: (Constant), DoV, Age, DoV_times_Age

Coefficients[a]

Model		Unstandardized Coefficients: B	Std. Error	Standardized Coefficients: Beta	t	Sig.	95.0% Confidence Interval for B: Lower Bound	Upper Bound	Correlations: Zero-order	Partial	Part
1	(Constant)	3.972E-7	.107		.000	1.000	-.220	.220			
	DoV	-.008	.015	-.106	-.531	.600	-.039	.023	-.106	-.106	-.106
2	(Constant)	-.008	.111		-.075	.941	-.237	.221			
	DoV	-.004	.015	-.055	-.272	.788	-.036	.028	-.106	-.057	-.054
	Age	-.016	.012	-.276	-1.364	.186	-.040	.008	-.289	-.274	-.271
	DoV_times_Age	.001	.002	.058	.292	.773	-.004	.006	.071	.061	.058

a. Dependent Variable: GlobCogPerf

S4.2 Regression GlobCogPerf versus DoV; modulators Age and (DoV * Age)

Patients with tracheostoma, n=24

Variables Entered/Removed[a]

Model	Variables Entered	Variables Removed	Method
1	DoV[b]	.	Enter
2	Age, DoV_CentAge[b]	.	Enter

a. Dependent Variable: GlobCogPerf
b. All requested variables entered.

Model Summary

Model	R	R Square	Adjusted R Square	Std. Error of the Estimate	Change Statistics				
					R Square Change	F Change	df1	df2	Sig. F Change
1	.413[a]	.170	.133	.7372076	.170	4.514	1	22	.045
2	.489[b]	.240	.125	.7402136	.069	.911	2	20	.418

a. Predictors: (Constant), DoV
b. Predictors: (Constant), DoV, Age, DoV_times_Age

ANOVA[a]

Model		Sum of Squares	df	Mean Square	F	Sig.
1	Regression	2.453	1	2.453	4.514	.045[b]
	Residual	11.956	22	.543		
	Total	14.409	23			
2	Regression	3.451	3	1.150	2.100	.132[c]
	Residual	10.958	20	.548		
	Total	14.409	23			

a. Dependent Variable: GlobCogPerf
b. Predictors: (Constant), DoV
c. Predictors: (Constant), DoV, Age, DoV_times_Age

Coefficients[a]

Model		Unstandardized Coefficients		Standardized Coefficients	t	Sig.	95.0% Confidence Interval for B		Correlations		
		B	Std. Error	Beta			Lower Bound	Upper Bound	Zero-order	Partial	Part
1	(Constant)	-8.333E-7	.150		.000	1.000	-.312	.312			
	DoV	.020	.010	.413	2.125	.045	.000	.040	.413	.413	.413
2	(Constant)	-.022	.153		-.143	.888	-.340	.297			
	DoV	.025	.010	.500	2.429	.025	.004	.046	.413	.477	.474
	Age	-.009	.015	-.126	-.616	.545	-.040	.022	-.101	-.137	-.120
	DoV_times_Age	.001	.001	.213	1.003	.328	-.001	.002	.096	.219	.195

a. Dependent Variable: GlobCogPerf

S4.3 Regression GlobCogPerf versus DoV; modulators CogRes and (DoV * Cogres)

Patients without tracheostoma, n=27.

Variables Entered/Removed[a]

Model	Variables Entered	Variables Removed	Method
1	DoV[b]	.	Enter
2	CogRes, DoV_CogRes[b]	.	Enter

a. Dependent Variable: CogPerf
b. All requested variables entered.

Model Summary

Model	R	R Square	Adjusted R Square	Std. Error of the Estimate	Change Statistics				
					R Square Change	F Change	df1	df2	Sig. F Change
1	.106[a]	.011	-.028	.5563103	.011	.282	1	25	.600
2	.683[b]	.467	.397	.4259059	.456	9.826	2	23	.001

a. Predictors: (Constant), DoV
b. Predictors: (Constant), DoV, centered_CogRes, DoV_times_CogRes

ANOVA[a]

Model		Sum of Squares	df	Mean Square	F	Sig.
1	Regression	.087	1	.087	.282	.600[b]
	Residual	7.737	25	.309		
	Total	7.824	26			
2	Regression	3.652	3	1.217	6.712	.002[c]
	Residual	4.172	23	.181		
	Total	7.824	26			

a. Dependent Variable: GlobCogPerf
b. Predictors: (Constant), DoV
c. Predictors: (Constant), DoV, CogRes, DoV_times_CentCogRes

Coefficients[a]

Model		Unstandardized Coefficients		Standardized Coefficients	t	Sig.	95.0% Confidence Interval for B		Correlations		
		B	Std. Error	Beta			Lower Bound	Upper Bound	Zero-order	Partial	Part
1	(Constant)	3.972E-7	.107		.000	1.000	-.220	.220			
	DoV	-.008	.015	-.106	-.531	.600	-.039	.023	-.106	-.106	-.106
2	(Constant)	.001	.082		.017	.987	-.168	.171			
	DoV	-.005	.014	-.060	-.332	.743	-.033	.024	-.106	-.069	-.051
	centered_CogRes	.077	.017	.683	4.433	.000	.041	.113	.664	.679	.675
	DoV_times_CogRes	-.002	.002	-.122	-.671	.509	-.006	.003	-.054	-.138	-.102

a. Dependent Variable: GlobCogPerf

S4.4 Regression GlobCogPerf versus DoV; modulators CogRes and (DoV * CogRes)

Patients with tracheostoma, n=24

Variables Entered/Removed[a]

Model	Variables Entered	Variables Removed	Method
1	DoV[b]	.	Enter
2	CogRes, DoV_times_CogRes[b]	.	Enter

a. Dependent Variable: GlobCogPerf
b. All requested variables entered.

Model Summary

					Change Statistics				
Model	R	R Square	Adjusted R Square	Std. Error of the Estimate	R Square Change	F Change	df1	df2	Sig. F Change
1	.413[a]	.170	.133	.7372076	.170	4.514	1	22	.045
2	.635[b]	.403	.313	.6560251	.232	3.891	2	20	.037

a. Predictors: (Constant), DoV
b. Predictors: (Constant), DoV, CogRes, DoV_times_CogRes

ANOVA[a]

Model		Sum of Squares	df	Mean Square	F	Sig.
1	Regression	2.453	1	2.453	4.514	.045[b]
	Residual	11.956	22	.543		
	Total	14.409	23			
2	Regression	5.802	3	1.934	4.494	.014[c]
	Residual	8.607	20	.430		
	Total	14.409	23			

a. Dependent Variable: GlobCogPerf
b. Predictors: (Constant), DoV
c. Predictors: (Constant), DoV, CogRes, DoV_times_CogRes

Coefficients[a]

		Unstandardized Coefficients		Standardized Coefficients			95.0% Confidence Interval for B		Correlations		
Model		B	Std. Error	Beta	t	Sig.	Lower Bound	Upper Bound	Zero-order	Partial	Part
1	(Constant)	-8.333E-7	.150		.000	1.000	-.312	.312			
	DoV	.020	.010	.413	2.125	.045	.000	.040	.413	.413	.413
2	(Constant)	-.002	.136		-.012	.991	-.284	.281			
	DoV	.016	.009	.328	1.791	.088	-.003	.035	.413	.372	.310
	CogRes	.067	.024	.489	2.789	.011	.017	.116	.543	.529	.482
	DoV_times_CogRes	.000	.001	.014	.077	.939	-.003	.003	.126	.017	.013

a. Dependent Variable: GlobCogPerf

S5 Detailed partial linear regression graphs

Figure S5 Averaged centered z scores versus each of the three independent variables

Including no IMV group and with the standard error of the mean (SEM) of the individual scores, The SEMs are shown for illustrative purposes only and were not included in the linear regression analyses.

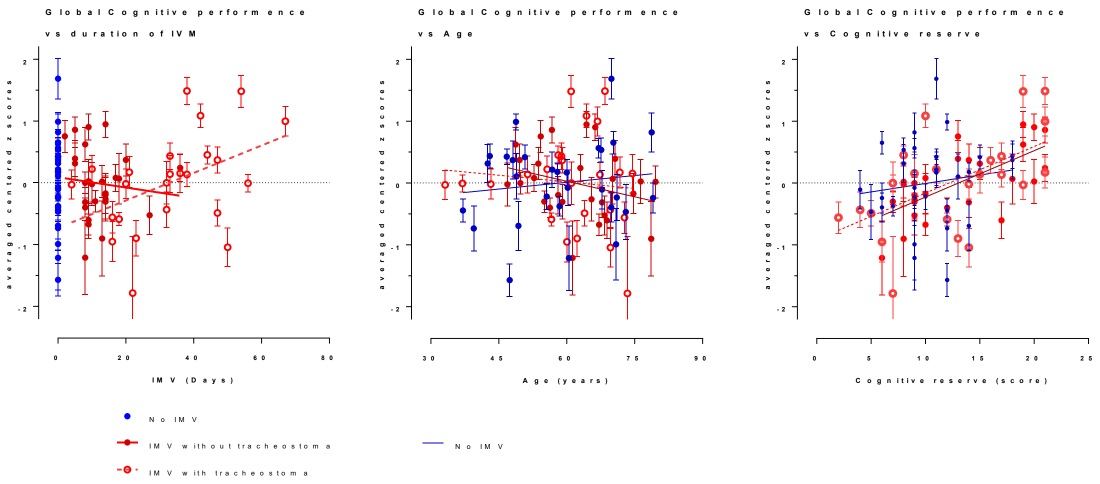

Table S5 Outcomes Partial linear regression outcomes

**Days of IMV**	IMV without tracheostoma	IMV with tracheostoma
Slope	-0.00832	0.02225
SE of fit Slope	0.00968	0.00514
F (DFn, DFd)	0.7382 (1, 187)	18.73 (1, 164)
P value	0.39130	<0.0001
r^2	0.00393	0.10250
Cohen's f² (R² / (1 - R²)	0.00395	0.11421

**Age**	No IVM	IMV without tracheostoma	IMV with tracheostoma
Slope	0.007295	-0.01677	-0.00714
SE of fit Slope	0.005967	0.00718	0.00784
F (DFn, DFd)	1.495 (1,200)	5.454 (1, 187)	0.8293 (1, 164)
P value	0.2229	0.02060	0.36380
r^2	0.007418	0.02834	0.00503
Cohen's f² (R² / (1 - R²)	0.00747	0.02917	0.00506

**Cognitive reserve**	No IVM	IMV without tracheostoma	IMV with tracheostoma
Slope	0.02725	0.07498	0.0753
SE of fit Slope	0.02025	0.01312	0.01351
F (DFn, DFd)	1.8801 (1,200)	32.67 (1,187)	31.05 (1,164)
P value	0.1801	<0.0001	<0.0001
r^2	0.008967	0.1487	0.1592
Cohen's f² (R² / (1 - R²)	0.00905	0.17467	0.18934

S6 Per domain Linear Regression, with and without tracheostoma

Figure S6 Per domain Linear Regression, with and without tracheostoma

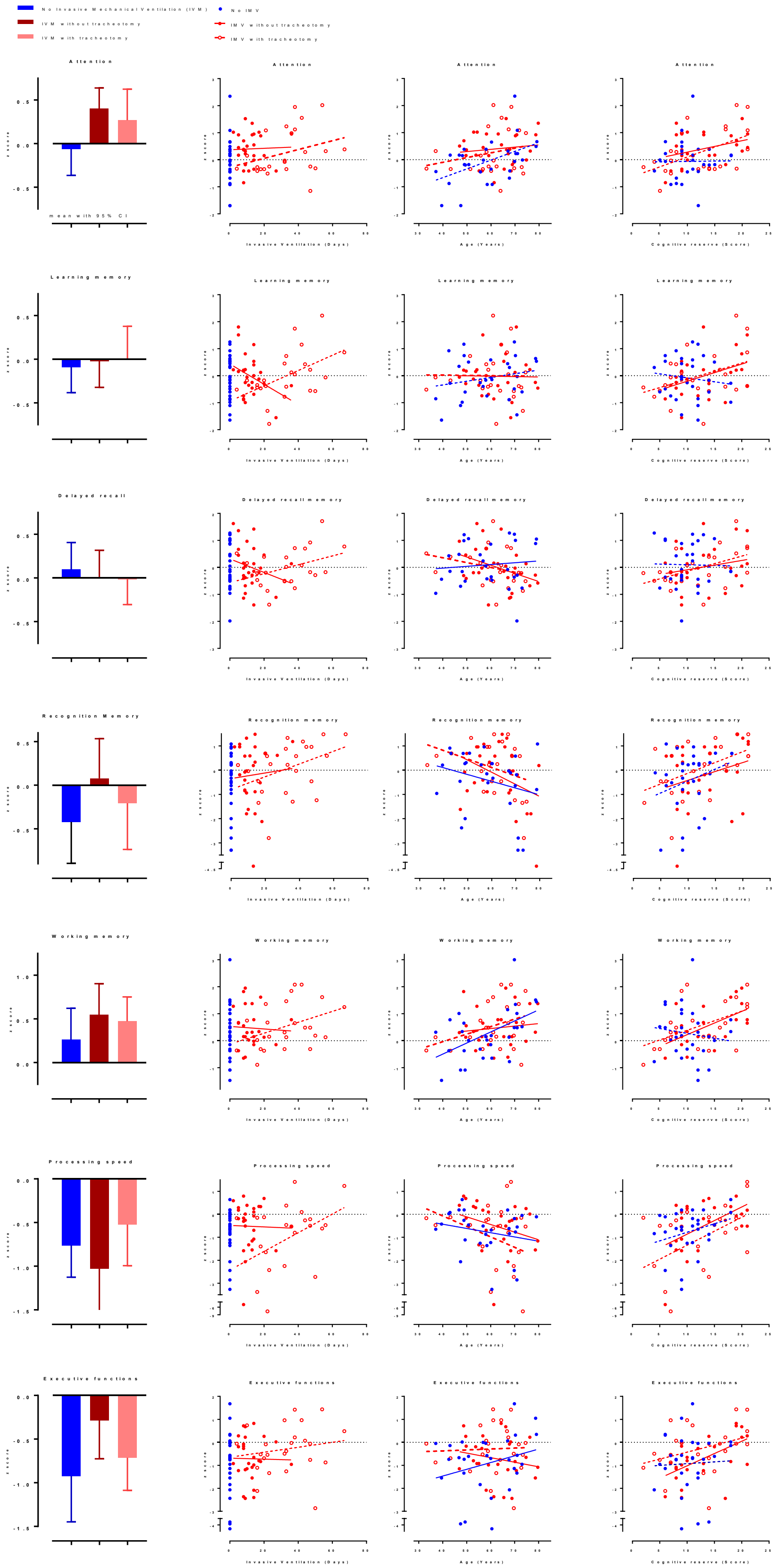

Table S6 Per domain Slopes of Linear Regression, with and without tracheostoma

Days of Ventilation	IMV without tracheostoma	IMV with tracheostoma
Attention	0.0027	0.0163
LearningMemory	-0.0374	0.0283
DelayedRecall	-0.0250	0.0165
RecognitionMemory	0.0116	0.0263
WorkingMemory	-0.0047	0.0202
ProcessingSpeed	-0.0033	0.0406
ExecutiveFunctions	-0.0021	0.0108
**mean**	-0.0083	0.0227
Lower 95% CI	-0.0240	0.0135
Upper 95% CI	0.0073	0.0319
One sample t test, t, df	t=1.301 df=6	t=6.0512 df=6
P value (two tailed)	0.2410	0.0009

Age	no IMV	IMV without tracheostoma	IMV with tracheostoma
Attention	0.0324	0.0082	0.0175
LearningMemory	0.0138	-0.0015	-0.0010
DelayedRecall	0.0066	-0.0302	-0.0178
RecognitionMemory	-0.0279	-0.0500	-0.0349
WorkingMemory	0.0410	0.0092	0.0279
ProcessingSpeed	-0.0184	-0.0332	-0.0455
ExecutiveFunctions	0.0293	-0.0199	0.0041
**mean**	0.0110	-0.0168	-0.0071
Lower 95% CI	-0.0132	-0.0378	-0.0321
Upper 95% CI	0.0351	0.0042	0.0178
One sample t test, t, df	t=1.1113 df=6	t=1.9526 df=6	t=0.6993 df=6
P value (two tailed)	0.3090	0.0987	0.5105

Cognitive reserve	no IMV	IMV without tracheostoma	IMV with tracheostoma
Attention	0.0026	0.0422	0.0728
LearningMemory	-0.0292	0.0637	0.0605
DelayedRecall	-0.0050	0.0346	0.0561
RecognitionMemory	0.0983	0.0729	0.0887
WorkingMemory	-0.0357	0.0862	0.0711
ProcessingSpeed	0.0742	0.1182	0.1216
ExecutiveFunctions	0.0153	0.1070	0.0608
**mean**	0.0172	0.0750	0.0759
Lower 95% CI	-0.0298	0.0461	0.0548
Upper 95% CI	0.0642	0.1039	0.0971
One sample t test, t, df	t=0.8973 df=6	t=6.3506 df=6	t=8.7835 df=6
P value (two tailed)	0.4041	0.0007	0.0001

S7 Delirium

Figure S7.1 For patients with and without delirium DoV, Age, CogRes and GlobCogPerf.

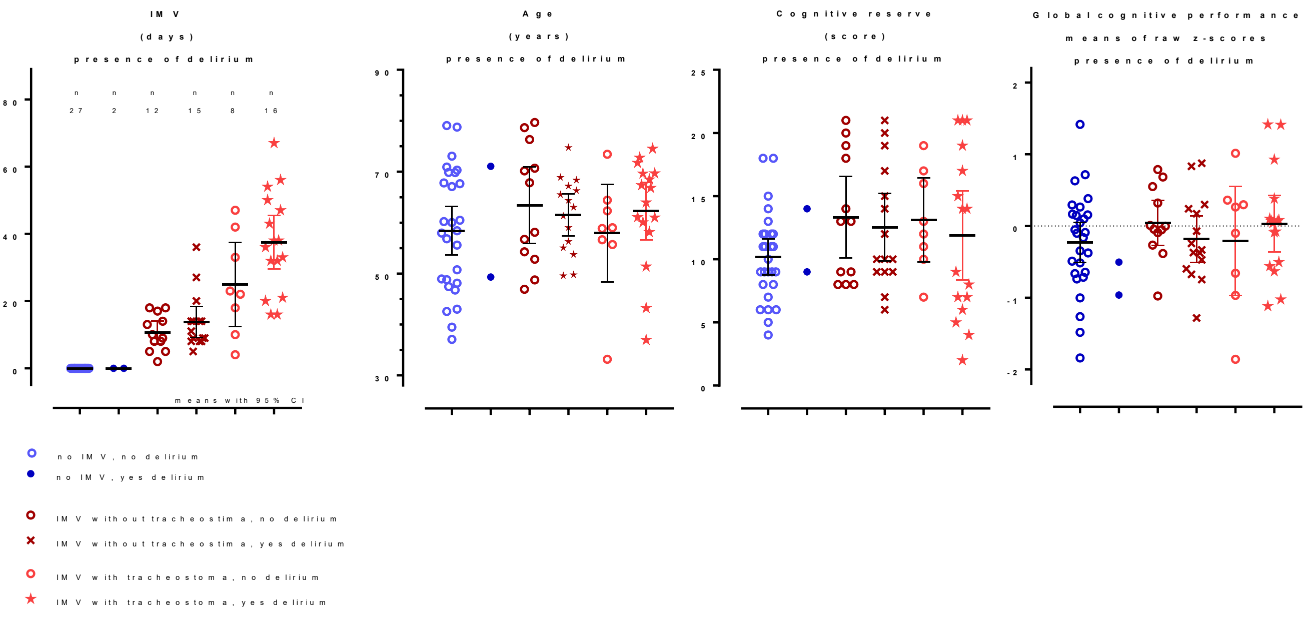

Figure S7.2 For patients with and without delirium: linear regression of GlobCogPerf vs. IMV days

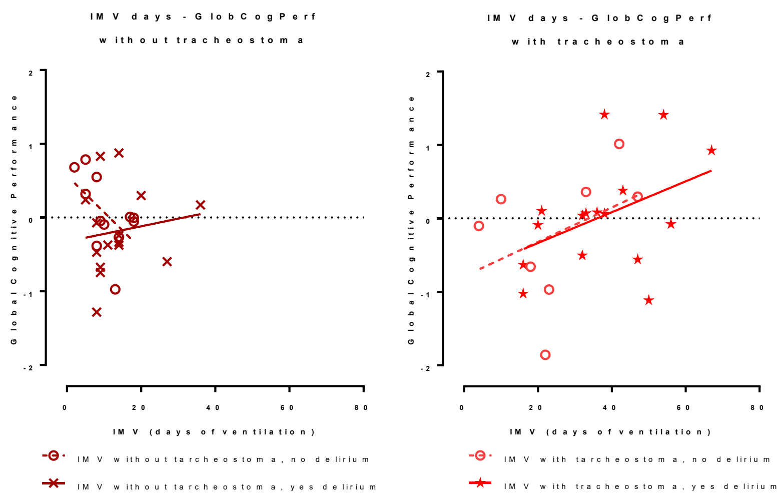

Supplement: Supplementary file 1 [file jcm-15-00898-s001.zip › S2_Rethinking Mechanical Ventilation_VanRijn et al_supplement 2_revised 1.pdf]
